# Individual Differences and Susceptibility to Burnout Syndrome: Sensory Processing Sensitivity and Its Relation to Exhaustion and Disengagement

**DOI:** 10.3389/fpsyg.2021.751350

**Published:** 2021-11-23

**Authors:** Krystyna Golonka, Bożena Gulla

**Affiliations:** Institute of Applied Psychology, Faculty of Management and Social Communication, Jagiellonian University, Kraków, Poland

**Keywords:** burnout, sensory processing sensitivity (SPS), highly sensitive person, Oldenburg Burnout Inventory (OLBI), Highly Sensitive Person Scale (HSPS), well-being

## Abstract

The aim of the study is to analyze the relationship between sensory processing sensitivity and occupational burnout. This analysis makes it possible to explore the problem of sensitivity as a predictor of burnout syndrome and to discuss adequate coping strategies in the work environment to improve employee well-being. The sample consists of 516 employees (236 women; *M*_*age*_ = 28.97, *SD* = 6.62 years). The *Highly Sensitive Person Scale* (HSPS; 27 items) was used to measure sensory processing sensitivity; the *Oldenburg Burnout Inventory* (OLBI; 16 items) was used to measure exhaustion and disengagement from work. The analysis of sensitivity is based on the ‘DOES’ model of a highly sensitive person, which includes *depth of processing* (D), *overstimulation* (O), *emotional reactivity and empathy* (E), and *sensing the subtle* (S). Burnout due to exhaustion and disengagement is analyzed. The factor analysis of the HSPS confirms its three-factor structure. The results indicate significant relationships between sensory processing sensitivity and burnout symptoms. However, the influence of specific aspects of sensitivity on the burnout problem varies: the results indicate that higher *emotional reactivity* is linked to worse burnout symptoms, but *sensing the subtle* shows the opposite effect and may be a protective factor against exhaustion. Significant gender differences were observed [*F*(511,1) = 136.63, *p* < 0.001; η^2^ = 0.21]; women revealed a significantly higher level of general sensitivity (*M*_women_ = 4.66, *SD* = 0.62) as compared to men (*M*_men_ = 4.01; *SD* = 0.64). Awareness of one’s high sensory-processing sensitivity and its potential outcomes in the work environment may be essential in order to implement appropriate regulatory strategies. Proactive strategies aimed at reducing prolonged emotional overload may be critical for highly sensitive employees. Recognizing high sensitivity may reduce burnout symptoms and improve well-being at work.

## Introduction

Research on job burnout has been carried out for nearly four decades, but in recent years it has become an extremely important topic. Changing work environments, rapidly developing technology, time pressure, globalization related to contact with different cultures and working in different time zones are all associated with high work demands and the necessity to constantly adapt to new job conditions. These trends, however, have been significantly reinforced since 2020 due to the COVID-19 pandemic. In an article entitled “How the Pandemic Exacerbated Burnout” (Lievens, 2021), the author interviewed pioneering burnout researchers Michael Leiter and Christina Maslach, both of whom emphasized that the pandemic has intensified the conditions that lead to burnout.

Burnout syndrome is defined as a psychological reaction to prolonged work-related stress that is influenced by both individual and organizational context (Schaufeli et al., 1993). This reaction has a processual nature and combines several components: (1) a feeling of energy depletion or exhaustion; (2) increased mental distance from one’s job, or a feeling of negativism or cynicism related to one’s job; and (3) reduced professional efficacy (World Health Organization, 2019). This assumption is based on the psychological definition of burnout syndrome introduced by Maslach et al. (1996), who described burnout as a state of exhaustion, depersonalization or cynicism, and low professional efficacy. According to the latest 11th International Classification of Diseases (ICD-11), burnout is an occupational phenomenon that is specifically related to stress at work that is not being effectively managed. It is not classified as a medical condition; however, it may lead to an increased risk of health problems. It is classified among ‘Factors influencing health status or contact with health services’ in the section ‘Problems associated with employment or unemployment’ (code: QD85) (World Health Organization, 2019).

In its more than 40-year history, the research on work-related chronic stress has focused on the antecedents, consequences and methods of counteracting burnout syndrome (Maslach and Leiter, 2016). The existing findings reveal that both organizational and individual factors are of great importance in the response to burnout. In this perspective, Leiter and Maslach (2004) accentuated the problem of mismatches between the individual and the work environment regarding workload, control, reward, community, fairness, and values. It is especially relevant to study individual differences when seeking characteristics that may be crucial in stress response and that influence a greater mismatch between an employee and their job. The benefits of research on burnout from the point of view of individual differences help to describe, explain and predict individual responses in particular work circumstances. Gaining knowledge in this area may help individuals understand the relation between their needs, abilities, limitations, values, and the work itself. On the other hand, selection, support and training for employees could be much more adequate if we understood the further consequences for well-being of employees’ characteristics that might cause them to be predisposed to overstimulation and exhaustion.

The research on factors that may predispose an employee to burnout syndrome emphasize the role of individual differences (e.g., Langelaan et al., 2006; Van der Linden et al., 2007). Research on the relationships between work stress and individual temperament has shown that temperamental variables were related to the perceived work stress in a group of general hospital nurses (Kikuchi et al., 2013). In terms of affective temperaments, Akiskal et al. (1998) found that depressive temperament was associated with over-commitment and a sense of a lack of balance between effort and reward. This temperament is characterized by the tendency to blame oneself, shyness, lack of assertiveness, and sensitivity to criticism. An anxious temperament is related to over-commitment. Kikuchi et al. (2013) suggest that nurses with a depressive or anxious temperament should be identified, monitored for all signs of job stress, and adequately supported by interventions that prevent adverse physical and mental effects. The findings of Kikuchi et al. (2013) indicate that temperamental characteristics may result in stressors being experienced as more serious than they actually are. The existing research implies that personality may moderate levels of experienced stress (Van der Linden et al., 2007) and influence stress-related consequences such as health problems and burnout (e.g., Cano-García et al., 2005). Research on personality traits emphasizes the role of neuroticism, negative affectivity, and anxiety as significant antecedents of burnout (e.g., Langelaan et al., 2006; Golonka et al., 2019). In this work, by referring to the concept of a highly sensitive person (Aron and Aron, 1997), we focus on sensory processing sensitivity as a temperamental characteristic in order to explore its association with employees’ exhaustion and disengagement.

### The Concept of a Highly Sensitive Person

The concept of the highly sensitive person relates to the Sensory Processing Sensitivity (SPS) construct, which is well known in the scientific literature. The high-sensitivity construct falls within a broader theoretical framework, known as environmental sensitivity, which assumes that humans can be classified according to the extent to which they register and process environmental stimulation (Greven et al., 2019). Regardless of the relationships with other psychological constructs, according to Pluess et al. (2018) the existing data indicate that high sensitivity is a separate construct. A highly sensitive person is an individual who distinguishes lower-intensity stimuli. This lower threshold of stimuli perception entails the tendency to become more easily distressed in response to higher levels of stimulation. The characteristics of a highly sensitive person include the tendency to process information more deeply; susceptibility to overstimulation due to greater sensitivity of the senses and more intense experiences; high emotional reactivity; and the ability to notice subtleties and nuances. The DOES acronym that describes highly sensitive persons stands for D – *depth of processing*, O – *overstimulation*, E – *emotional reactivity and empathy*, S – *sensing the subtle* (Aron, 2013).

Before the concept of high sensitivity appeared, individuals with such dispositions were described as shy, insecure, anxious, introvert or neurotic. In common knowledge, these terms refer to features that have low social value and may be pejorative. When high sensitivity is described in terms of specific characteristics, this normalizes possible states, e.g., tiredness, exhaustion, irritation, frustration, as natural consequences of being overstimulated. Furthermore, it helps to emphasize the resources and valuable features of a highly sensitive person, e.g., empathy. The theme of a positive view of people who may sometimes be less effective, a bit withdrawn, less open or more easily overwhelmed by stimuli is accentuated by Wagele (2006) and Cain (2012), who show “the power of introverts.”

Research findings suggest an association between SPS and personality traits and between SPS and positive and negative affect (e.g., Lionetti et al., 2019; Bröhl et al., 2020). When trying to define the relationship between SPS and the Big Five personality traits, Lionetti et al. (2019) adopted the three-factor solution of SPS proposed by Smolewska et al. (2006): *Ease of Excitation* – EOE, *Aesthetic Sensitivity* – AES, and *Low Sensory Threshold* – LST. A meta-analysis based on 24 selected articles revealed that sensory processing sensitivity is to some extent related to other individual characteristics that reflect environmental sensitivity, such as introversion, neuroticism, openness, behavioral inhibition and negative affect. However, the data on these relationships were not always consistent, especially when the SPS subscales were included. With regard to the Big Five dimensions (8 articles; 6,790 participants), SPS in children correlated with neuroticism (*r* = 0.42) but not with extraversion, openness, agreeableness or conscientiousness. In adults, SPS correlated with neuroticism (*r* = 0.40) and openness (*r* = 0.14), but not with extraversion, agreeableness, or conscientiousness. With regard to positive and negative affect (19 studies; 5,326 participants), SPS in children was moderately correlated with both negative (*r* = 0.29) and positive (*r* = 0.21) affect, but only with negative affect (*r* = 0.34) in adults. The authors conclude that the relationships between SPS and personality traits and affect are complex configurations, and SPS is relatively distinct from other personality traits and affect in both children and adults. Bröhl et al. (2020) observed that for sensory processing sensitivity the most relevant variables were neuroticism and openness to experience. Extraversion was less related to sensory processing sensitivity, while the relationships between SPS and conscientiousness and agreeableness were of little importance. In older adolescents and young adults, openness was significantly associated with the *Aesthetic Sensitivity* subscale, which relates to aesthetic experiences, creativity and cognitive curiosity. In contrast, anxiety and lack of self-confidence were linked with the *Ease of Excitation* and *Low Sensory Threshold* subscales. According to these authors, in order to capture the relationships between SPS and various dimensions of personality, it is necessary to analyze these relationships at the level of specific aspects of sensory processing sensitivity.

Sensitivity relates to temperamental characteristics; it has a biological basis and distinct neurobiological correlates. Symptoms of a highly sensitive central nervous system are already noticeable in reactions to stimuli in infancy (Belsky and Pluess, 2009), and childhood (Boterberg and Warreyn, 2016), and they are combined with strong emotional reactions and empathy. Belsky and Pluess (2009) highlighted the importance of plasticity in human development in the context of varying vulnerability: some children with negatively emotional temperaments or certain genotypes are more susceptible to the effects of negative experiences, but they may also be more prone to positive experiences, which are less frequently analyzed in psychology. Assary et al. (2020) showed that differences in susceptibility to external influences and ways of reacting to the environment have a genetic basis. Using the *Highly Sensitive Child Scale*, in a large sample of adolescent twins the authors determined that the heritability of sensitivity was 0.47. They found that the genetic factors that underlie sensitivity to positive environmental influences are different from those underlying sensitivity to negative environmental ones. This supports the concept of a multi-dimensional genetic model of environmental sensitivity. Furthermore, Assary et al. (2020) identified common and specific genetic and environmental influences on the level of each aspect of environmental sensitivity (EOE, AES, LST). The latent sensitivity factor, which relates to common genetic and environmental influences, was heritable (0.51) and explained 90% of ease of excitation variance, 58% of low sensory threshold, and 29% of aesthetic sensitivity. Assary et al. (2020) indicate that the phenotypic similarities regarding environmental sensitivity, neuroticism and extraversion can largely be explained by common genetic influences, while the differences can be attributed to unique environmental influences.

Chen et al. (2011) and Homberg et al. (2016) indicate the genetically determined nature of the HSP disposition: high sensitivity is combined with genetically determined levels of selected neurotransmitters, i.e., lower availability of dopamine and serotonin. Acevedo et al. (2014) showed that highly sensitive persons manifest higher activation of mirror neurons, which are associated with empathy and imitation. Additionally, highly sensitive participants revealed higher activation in the insula. This brain structure is described as a center of sensory awareness; it is associated with limbic functions and self-referential processing (Fan et al., 2011; Acevedo et al., 2014). High sensory processing sensitivity is associated with activation of the brain in regions responsible for deep processing, memory and psychophysiological regulation in response to emotive stimuli (Acevedo et al., 2017, 2021). Using functional magnetic resonance imaging (fMRI), Acevedo et al. (2017) analyzed brain activity due to sensory processing sensitivity exposure to negative, positive or neutral stimuli revealed that highly sensitive sensory processing was related to increased activation of the hippocampus, the hypothalamus, the entorhinal area, and the temporal gyri; it was also related to decreased activation of the inferior parietal area. Interestingly, these researchers analyzed the influence of early environmental impacts on neural response to emotional stimuli. Positive subjective assessment of one’s own childhood was associated with lower intensity of response to stimuli in certain brain areas. The positive childhoods of highly sensitive people were found to be related to activation of reward areas, which act as a buffer in emotional situations; on the other hand, negative childhoods were associated with activation of the amygdala, which is associated with responses to threats. A positive childhood may favor adaptation to emotogenic stimuli through the higher-order cortical systems involved in consciousness, reflexive thinking and self-regulation. In the light of neuroimaging research, it may be assumed that sensory processing sensitivity has a biological basis and relates to the functioning of the central nervous system; however, life experiences may modulate HSPs’ responses to emotogenic stimuli and therefore influence the types of regulating strategies that these persons apply.

**FIGURE 1:**
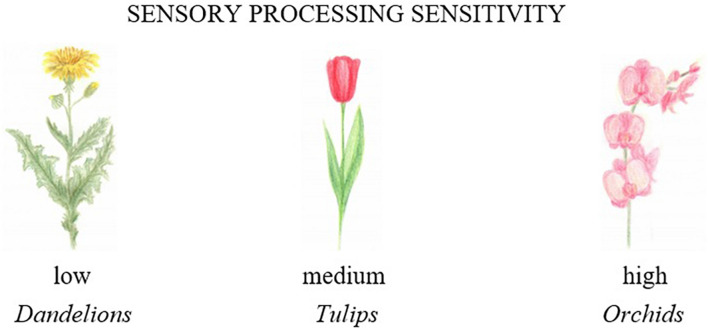
Different levels of sensory processing sensitivity – flower metaphor (Boyce and Ellis, 2005; Lionetti et al., 2018).

Borries and Ostendorf (2012) indicated that sensitivity may not be a dimension but rather a category, and emphasized its dichotomic nature (either one is highly sensitive or not). However, further research on the sensitivity of sensory processing instead designates this disposition as a continuum in which three main levels of sensitivity are indicated: low, medium and high (Lionetti et al., 2018; Greven et al., 2019). The flower metaphor, introduced by Boyce and Ellis (2005), who called highly sensitive individuals (highly reactive phenotypes) *Orchids* and low-sensitive individuals (low-reactive phenotypes) *Dandelions*, was further developed by Lionetti et al. (2018), who introduced a third group of moderately sensitive people, known as *Tulips*. *Dandelions* represent people who do not need special circumstances in order to function; *Tulips* (medium sensitivity) have moderate needs and requirements; and *Orchids* represent people who need special circumstances in which they can fully function, develop and use their potential. This metaphoric attitude is presented in Figure 1.

Summarizing, high sensitivity is an individual characteristic; it is neither a weakness nor a defect. An HSP does not need protection; however, this person does need support and proper conditions that will enable them to use their abilities and potential. It is especially important to learn how to function efficiently with the disposition of high sensory processing sensitivity. Aron (2013) compares high sensitivity to left-handedness: most people are right-handed, but this does not mean that there is something is wrong with being left-handed.

Grimen and Diseth (2016) observed that some aspects of sensory processing sensitivity are associated with subjective health complaints. Evers et al. (2008) dealt with the importance of high sensory processing sensitivity in relation to work-related stress and the risk of burnout. A highly sensitive person may perceive the workplace as being full of strong, chaotic stimuli; this causes difficulties in understanding and carrying out tasks and managing their own functioning in the workplace. As a further consequence, work ceases to be a source of satisfaction; it becomes a reason for feeling overloaded; it inflicts emotional distress; it leads to dissatisfaction with work that results in a need for change. Evers et al. (2008) showed that high sensitivity measured by HSPS is associated with a high level of work stress; however, this relationship is not visible in the first phase of the stress response (the alarm phase) as in this stage a sense of work overload or emotional burden is not observed. However, dissatisfaction with work and the need to make changes was identified in the second phase of the stress response (the adaptation stage). This indicates the increased risk of burnout in highly sensitive individuals who, depending on their resources, initially seem to meet job demands but may bear higher psychological costs in the long term.

Most of the research models on burnout explore the role of individual traits in developing burnout syndrome. In this work, we will analyze the relationship between sensory processing sensitivity and occupational burnout. Knowledge of the link between burnout and sensitivity may facilitate regulatory behaviors and proper coping strategies, both of which may influence employees’ well-being. Furthermore, we will discuss the possible practical implications for both employees and organizations, thus enabling them to support highly sensitive persons.

## Materials and Methods

### Participants and Procedure

The sample consists of 516 adult employees (236 women: 45.7%; 277 men: 53.7%; 3 did not state: 0.06%). The average age of participants was 28.97 years (*SD* = 6.62). The sample consists of various employee groups, but the largest groups represented IT (26%) and financial (13%) companies. 10% of the sample held managerial positions in small (up to five persons: 42% of managers) and big teams (more than 25 persons: 20% of managers). The 76% of respondents worked full time. Most of the participants had completed higher education (74%).

Regarding the other sociodemographic data, 67% of respondents were married or in relationships; 20% had at least one child. Only 8% of the respondents did not answer the open question concerning the potential sources of stress besides work. Most frequently, answers referred to family duties and responsibilities, the need to combine work with studies, and health problems. 6% of the sample reported serious health problems; 20% some health problems; 20% assessed their health as average; most of the sample (57%) assessed their health as good or very good. Many of the tested employees revealed sleep problems. Only 24% of the sample reported very good quality of sleep; other respondents complained about problems with falling asleep (8.5%), waking up at night (11%), or early awakening (27%). The 34% of respondents found their sleep not fully regenerative, and 26% complained of sleep deficit.

This online study was voluntary; it was conducted in accordance with the recommendations of the Helsinki declaration and was accepted by the Research Ethics Committee of the Institute of Applied Psychology, Jagiellonian University in Kraków.

### Methods

Two self-report instruments were used in the study: the *Highly Sensitive Person Scale* (HSPS) and the *Oldenburg Burnout Inventory* (OLBI).

*Highly Sensitive Person Scale* was developed by Aron and Aron (1997). The Polish version was developed by the authors of this article using a back translation procedure. The questionnaire consists of 27 items answered on a seven-point scale from 1 (*not at all*) to 7 (*extremely*). The analysis proposed in this paper refers to the three-factorial solution that was confirmed in two studies by Smolewska et al. (2006), and Evers et al. (2008): EOS (*Ease of Excitation*, 12 items), AES (*Aesthetic Sensitivity*, 6 items) and LST (*Low Sensory Threshold*, 7 items). The three-factor structure of the HSPS has been confirmed in other studies, e.g., Grimen and Diseth (2016), Lionetti et al. (2018). However, results of Lionetti et al. (2018) suggest three orthogonal scales and one general sensitivity factor across all items. Some research specifies a different HSPS structure: there are also one-factor (Aron and Aron, 1997), two-factor (Evans and Rothbart, 2008; Ershova et al., 2018; Montoya-Pérez et al., 2019), five-factor (Chacón et al., 2021), and even six-factor (Blach, 2016) solutions. In Evans and Rothbart’s (2008) research, in which the HSP scale was compared with the *Adult Temperament Questionnaire* (ATQ), two factors were distinguished: the first covers negative emotionality and is close to the neuroticism construct (high correlations with *Negative Affect* and its sensory discomfort subscale in ATQ); the second factor concerns paying attention to external and internal stimuli (high correlations with *Orienting Sensitivity* and its sensory sensitivity subscale in ATQ). HSPS demonstrated strong reliability across samples: Cronbach’s alpha higher than 0.84 (May et al., 2020).

*The Oldenburg Burnout Inventory* was developed by Demerouti et al. (2001) and Demerouti and Bakker (2008). Baka and Basińska’s (2016) Polish adaptation of OLBI was used in this study. The questionnaire contains 16 items, rated on a scale from 1 (*agree*) to 4 (*disagree*). The analyses confirmed that the structure of the questionnaire is based on two subscales: exhaustion and disengagement from work. The method includes positively and negatively worded items. Example items for exhaustion: “After my work, I regularly feel worn out and weary” and “After my work, I regularly feel totally fit for my leisure activities” (reversed). Example items for disengagement from work: “I frequently talk about my work in a negative way,” and “I get more and more engaged in my work” (reversed) (Demerouti and Bakker, 2008). The results are summed up separately for each subscale, but the most common factor is the overall burnout factor, which is the sum of all items. This method has satisfactory psychometric parameters: Cronbach’s alpha for individual scales ranges from 0.82 to 0.89; for the general indicator, the coefficient was 0.88. The analyses showed that both exhaustion and disengagement correlated positively with perceived stress and negatively with commitment to work (Baka and Basińska, 2016).

## Results

The analysis was conducted using SPSS version 27 (IBM SPSS Statistics, IBM Corporation, United States) with the Proces_v3.5 module (Hayes, 2018). The analyses included a factor analysis for HSPS to describe the structure of the Polish version of the scale. The k-means cluster analysis was conducted in order to define potential clusters that are differentiated in terms of the level of sensory processing sensitivity. Following Lionetti et al. (2018), we ran a cluster analysis with three defined clusters. To explore the characteristics of each cluster, mean difference tests on sensitivity features and burnout symptoms were performed. Then, the correlation analysis was conducted to explore the relationships between burnout symptoms and sensory processing sensitivity and its specific factors. The regression analysis provided insight into which aspects of SPS are significant for particular burnout symptoms. Additional analyses were dedicated to investigating gender differences because previous findings have shown significantly higher sensitivity scores among women (e.g., Jonsson et al., 2014; Şengül-l̇nal and Sümer, 2020) and significant differences in burnout symptoms between men and women (Ahola et al., 2006).

A principal components analysis with oblimin oblique rotation was applied to test the three-factorial solution for HSPS as it was presented by Smolewska et al. (2006; Table 1). These three components accounted for 37% of the variance (eigenvalues: 6.35, 2.10, 1.51). Individual items were included as component indicators if the loading on a given component was greater than 0.35. If an item loaded high on more than one component, it was eliminated from further analyses. The analysis of the Polish version of HSPS led to the elimination of two items: item 6 – *Are you particularly sensitive to the effects of caffeine?*; and item 24 – *Do you make it a high priority to arrange your life to avoid upsetting or overwhelming situations?* Item 6 loaded below 0.35; item 24 loaded high on two components: *Emotional Reactivity* and *Overstimulation*. To analyze the general indicator of HSPS, all items from the original version of the scale were included. The analysis confirmed the structure of HSPS as it was presented by Smolewska et al. (2006). However, we propose labeling each component differently than was done in previous studies (Smolewska et al., 2006; Evers et al., 2008) by referring to the DOES model (Aron, 2013) and considering the content of the items included in each component. The components are defined as three HSPS subscales: (1) *Emotional Reactivity* (ER); (2) *Sensing the Subtle* (StS); (3) *Overstimulation* (OvSt).

**TABLE 1:** Principal components analysis with oblimin rotation: Component loadings, alphas, and mean inter-item correlations for the HSPS (three-factor analysis).

**Item**		**Components**		
		**I (ER)**	**II (StS)**	**III (OvSt)**
1.	Are you easily overwhelmed by strong sensory input?	0.70		
3.	Do other people’s moods affect you?	0.51		
4.	Do you tend to be more sensitive to pain?	0.48		
5.	Do you find yourself needing to withdraw during busy days into bed or into a darkened room or any place where you can have some privacy and relief from stimulation?	0.56		
11.	Does your nervous system sometimes feel so frazzled that you just have to go off by yourself?	0.54		
13.	Do you startle easily?	0.54		
14.	Do you get rattled when you have a lot to do in a short amount of time?	0.69		
16.	Are you annoyed when people try to get you to do too many things at once?	0.61		
21.	Do changes in your life shake you up?	0.60		
23.	Do you find it unpleasant to have a lot going on at once?	0.63		
26.	When you must compete or be observed while performing a task, do you become so nervous or shaky that you do much worse than you would otherwise?	0.59		
27.	When you were a child, did parents or teachers seem to see you as sensitive or shy?	0.56		
2.	Do you seem to be aware of subtleties in your environment?		0.49	
8.	Do you have a rich, complex inner life?		0.59	
10.	Are you deeply moved by the arts or music?		0.72	
12.	Are you conscientious?		0.30	
15.	When people are uncomfortable in a physical environment, do you tend to know what needs to be done to make it more comfortable (like changing the lighting or the seating)?		0.53	
22.	Do you notice and enjoy delicate or fine scents, tastes, sounds, works of art?		0.73	
7.	Are you easily overwhelmed by things like bright lights, strong smells, coarse fabrics, or sirens close by?			0.55
9.	Are you made uncomfortable by loud noises?			0.75
17.	Do you try hard to avoid making mistakes or forgetting things?			0.40
18.	Do you make a point to avoid violent movies and TV shows?			0.55
19.	Do you become unpleasantly aroused when a lot is going on around you?			0.60
20.	Does being very hungry create a strong reaction in you, disrupting your concentration or mood?			0.43
25.	Are you bothered by intense stimuli, like loud noises or chaotic scenes?			0.81
	Coefficient alpha	0.83	0.61	0.74
	Mean inter-item correlation	0.30	0.20	0.30

*ER, *Emotional Reactivity*; StS, *Sensing the Subtle*; and OvSt, Overstimulation.*

The Kolmogorov–Smirnov tests revealed that burnout symptoms did not meet the assumption of normality: *p-*value < 0.001 (for both exhaustion and disengagement). The normality of the distribution of sensory processing sensitivity was accepted with a *p*-value of 0.14 (5% significance level). In further analyses, the following statistical analyses were used: Spearman’s test for bivariate correlation; Kruskal–Wallis tests and Mann–Whitney *U* tests to examine the mean differences between clusters and gender, respectively.

### Cluster Analysis

**FIGURE 2:**
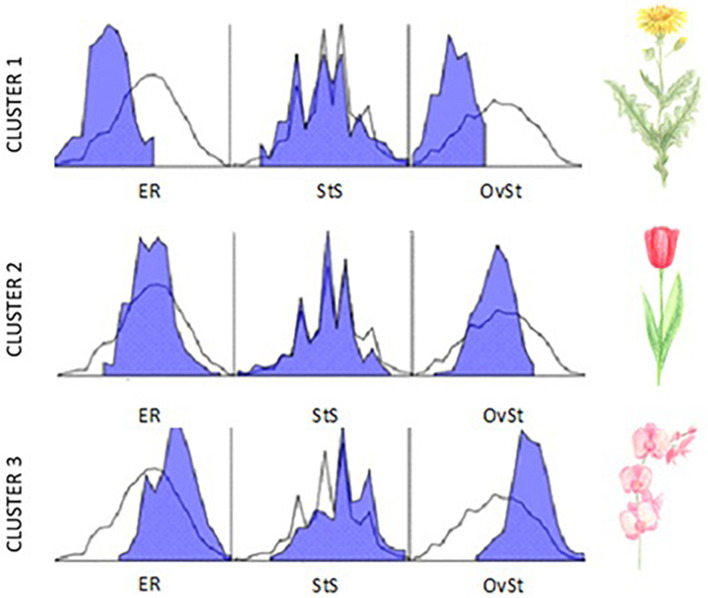
The HSPS subscales’ distribution in each cluster (axis *X* – scores in each subscale; axis *Y* – frequency; blue area presents the distribution for the cluster; white area presents the distribution for the general sample). ER, *Emotional Reactivity*; StS, *Sensing the Subtle*; OvSt, *Overstimulation*.

The *k*-mean cluster analysis, which was defined for three clusters (low, medium, high sensitivity), was run for the three HSPS subscales: (1) ER – *Emotional Reactivity*; (2) StS – *Sensing the Subtle* (StS); (3) OvSt – *Overstimulation*. The ANOVA results are reported in Table 2. The names of the clusters refer to the flower metaphor introduced by Boyce and Ellis (2005) and Lionetti et al. (2018): *Dandelions* (low sensitivity), *Tulips* (medium sensitivity), and *Orchids* (high sensitivity). Table 3 shows the differences in HSPS and each subscale’s mean scores between clusters, and the number of observations in each cluster. Figure 2 demonstrates the distributions of results in each HSPS subscale in the tested clusters.

**TABLE 2:** K-Means cluster groups with ANOVA results for the three subscales of HSPS.

	**Cluster**		**Error**		***F***	***p***
	**Mean square**	**df**	**Mean square**	**df**		
ER	124.433	2	0.364	513	342.287	<0.001
StS	17.090	2	0.542	513	31.556	<0.001
OvSt	202.638	2	0.319	513	635.115	<0.001

*ER, *Emotional Reactivity*; StS, *Sensing the Subtle*; and OvSt, *Overstimulation*.*

**TABLE 3:** Descriptive statistics M(SD) and number of observations for HSPS in the three clusters of sensitivity.

**Sensory processing sensitivity**	** 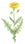 **	** 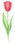 **	** 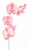 **
	**Low**	**Medium**	**High**
HSPS	3.35 (0.41)	4.19 (0.30)	5.03 (0.38)
ER	3.12 (0.58)	4.24 (0.65)	5.05 (0.58)
StS	4.59 (0.79)	4.54 (0.71)	5.10 (0.73)
OvSt	2.67 (0.56)	3.85 (0.57)	5.09 (0.56)
Observations	*n* = 107 (21%)	*n* = 232 (45%)	*n* = 177 (34%)

*HSPS, *Highly Sensitive Person Scale*; ER, *Emotional Reactivity*; StS, *Sensing the Subtle*; OvSt, Overstimulation.*

Table 4 shows the results of the Kruskal–Wallis test (*H* test) of difference between independent samples, which revealed that the clusters differ significantly in the mean of general HSPS scores [*H*(2) = 408.22, *p* < 0.001, η^2^ = 0.79]. In the general HSPS scores, each pair of compared clusters significantly differed from each other (*p* < 0.001). In the comparisons between clusters, in particular the HSPS subscales, the only insignificant difference was observed between *Dandelions* and *Tulips* in the *Sensing the Subtle* subscale.

**TABLE 4:** The results of the Kruskal–Wallis test on general sensitivity (HSPS scores) and HSPS subscales: *Emotional Reactivity*, *Sensing the Subtle*, and *Overstimulation*.

**Sample 1–Sample 2**	***n***	**K–W test**	**Error standardized**	**Standardized K–W test**	***p***	**η*^2^***	Effect size*
**HSPS scores**							

*Dandelions–Tulips*	339	–161.139	17.421	–9.250	<0.001	0.55	Large
*Dandelions–Orchids*	284	–358.599	18.255	–19.644	<0.001	0.70	Large
*Tulips–Orchids*	409	197.460	14.878	13.272	<0.001	0.66	Large

**Emotional reactivity**							

*Dandelions–Tulips*	339	–170.148	17.416	–9.769	<0.001	0.45	Large
*Dandelions–Orchids*	284	–309.628	18.250	–16.966	<0.001	0.67	Large
*Tulips–Orchids*	409	139.481	14.874	9.378	<0.001	0.31	Large

**Sensing the subtle**							

*Dandelions–Tulips*	339	3.712	17.381	0.214	0.831	0.00	–
*Dandelions–Orchids*	284	106.117	14.844	7.149	<0.001	0.10	Medium
*Tulips–Orchids*	409	–102.404	18.214	–5.622	<0.001	0.13	Medium

**Overstimulation**							

*Dandelions–Tulips*	339	–156.009	17.409	–8.962	<0.001	0.48	Large
*Dandelions–Orchids*	284	–345.066	18.242	–18.916	<0.001	0.70	Large
*Tulips–Orchids*	409	189.056	14.867	12.716	<0.001	0.58	Large

*Comparisons between pairs of clusters (*N* = 516).*

*Interpretation of η^2^: 0.01 – small, 0.06 – medium; 0.14 – large (Cohen, 1988).

Further analysis explored the relationship between sensitivity and burnout symptoms. The findings show that general sensitivity positively correlates with burnout symptoms: *Exhaustion* (*r*_*S*_ = 0.33, *p* < 0.001) and *Disengagement* (*r_*S*_* = 0.19, *p* < 0.001). However, investigating the different aspects of sensitivity led to an interesting observation: the strongest correlation was observed between *Emotional Reactivity* and *Exhaustion* (*r*_*S*_ = 0.42, *p* < 0.001) and *Disengagement* (*r*_*S*_ = 0.27, *p* < 0.001). A significant but weak positive correlation was detected between *Overstimulation* and both burnout symptoms (Table 5). Interestingly, the third HSPS subscale, *Sensing the Subtle*, shows the opposite direction: it negatively correlates with *Exhaustion* (*r*_*S*_ = −0.11, *p* < 0.05). Although this is a weak correlation, it shows a different pattern and a possible ‘protective’ aspect of sensitivity.

**TABLE 5:** Descriptive statistics, Cronbach alpha, and the results of Spearman’s rho correlation between the scores of the Oldenburg Burnout Inventory (OLBI) and the Highly Sensitive Person Scale (HSPS).

	**Variable**	***M***	***SD***	**α**	**1**	**2**	**3**	**4**	**5**	**6**
										
	**OLBI**									
1	Exhaustion	2.36	0.45	0.77	–					
2	Disengagement	2.35	0.45	0.70	0.52***	–				
3	**HSPS**	4.30	0.71	0.85	0.33***	0.19***	–			
4	ER	4.29	0.92	0.83	0.42***	0.27***	0.87***	–		
5	StS	4.74	0.78	0.61	−0.11*	−0.09	0.43***	0.15**	–	
6	OvSt	4.03	1.05	0.74	0.19***	0.16***	0.82***	0.56***	0.24***	–

*ER, Emotional Reactivity; StS, Sensing the Subtle; OvSt, Overstimulation (N = 516).*

*p < 0.05; **p < 0.01; ***p < 0.001.

Table 6 shows the statistics for the regression models for burnout symptoms where exhaustion and disengagement are explained variables and components of sensitivity: *Emotional reactivity*, *Sensing the Subtle*, and *Overstimulation*, are the tested predictors. Multiple regression analysis revealed that burnout symptoms may be explained by some aspects of sensory processing sensitivity. The regression model for exhaustion with three components of sensitivity is significant [*F*(3,512) = 47.84, *p* < 0.001] and shows that *Emotional Reactivity* and *Sensing the Subtle* are important predictors for exhaustion (*p* < 0.001 and *p* < 0.01, respectively). The model explains 21% of the variance of the exhaustion outcomes. The regression model for disengagement with three components of sensitivity (ER, StS, OvSt) is weaker but significant [*F*(3,512) = 12.66, *p* < 0.001] and shows a similar pattern to *Emotional Reactivity* and *Sensing the Subtle* as important predictors of disengagement (*p* < 0.001 for both components). The model explains 6% of the variance of the disengagement outcomes.

**TABLE 6:** Multiple regression analysis for variables predicting exhaustion and disengagement (*N* = 516).

**Model for exhaustion**		***B***	***SE***	**Beta**	***t***	***p***	**95% confidence interval of the *B***		**Statistics of multicollinearity**	
	**Variables**						**Lower**	**Upper**	**Tolerance**	**VIF**
	Constant	1.848	0.127		14.591	<0.001	1.599	2.097		
	ER	0.243	0.024	0.501	10.307	<0.001	0.197	0.290	0.645	1.551
	StS	–0.085	0.023	–0.149	–3.691	<0.001	–0.130	–0.040	0.942	1.062
	OvSt	–0.031	0.021	–0.073	–1.463	0.144	–0.072	0.011	0.620	1.612

*R* = 0.468, *R*^2^ = 0.219, Adj. *R*^2^ = 0.214										
**Model for disengagement**		***B***	***SE***	**Beta**	***t***	***p***	**95% confidence interval of the *B***		**Statistics of multicollinearity**	
	**Variables**						**Lower**	**Upper**	**Tolerance**	**VIF**

	Constant	2.157	0.139		15.552	<0.001	1.885	2.430		
	ER	0.113	0.026	0.233	4.382	<0.001	0.063	0.164	0.645	1.551
	StS	–0.073	0.025	–0.127	–2.901	0.004	–0.123	–0.024	0.942	1.062
	OvSt	0.013	0.023	0.029	0.545	0.586	–0.033	0.058	0.620	1.612

*R* = 0.263, *R*^2^ = 0.69, Adj. *R*^2^ = 0.064										

*VIF, variance inflation factor.*

**FIGURE 3:**
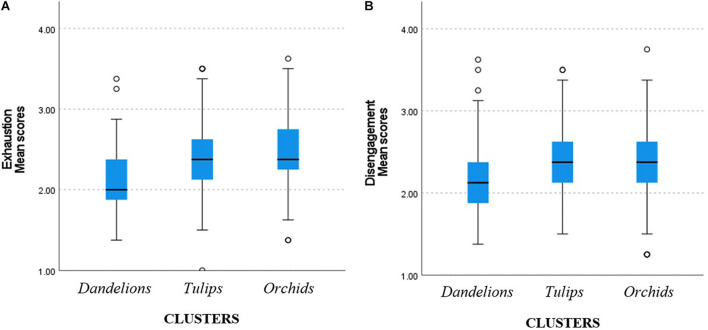
Mean scores on burnout symptoms in clusters of low (*Dandelions*), medium (*Tulips*), and high (*Orchids*) sensitivity: **(A)** *Exhaustion*; **(B)** *Disengagement*.

When analyzing the differences between clusters (low, medium, and high sensitivity), significant differences were detected in both burnout symptoms: exhaustion and disengagement (Figure 3).

The Kruskal–Wallis test revealed that there were significant differences between the tested groups in the level of exhaustion with medium effect of 7% explained variance [*H*(2) = 40.35, *p* < 0.001, η^2^ = 0.07]. Examination of the group means suggests that compared to medium and high sensitivity, the lowest sensitivity shows the lowest level of exhaustion. When investigating the differences between the clusters in terms of the level of disengagement, similar findings were observed: the Kruskal–Wallis test showed significant differences between groups with small effect of 4% explained variance [*H*(2) = 21.64, *p* < 0.001, η^2^ = 0.04]; as compared to medium and high sensitivity, the lowest sensitivity shows the lowest level of disengagement (Table 7).

**TABLE 7:** The results of Kruskal–Wallis test on exhaustion and disengagement: Comparisons between pairs of clusters (*N* = 516).

**Sample 1–Sample 2**	***n***	**K–W test**	**Error standardized**	**Standardized K–W test**	***p***	**η*^2^***	Effect size*
**Exhaustion**							

*Dandelions–Tulips*	339	–80.733	17.358	–4.651	<0.000	0.07	Medium
*Dandelions–Orchids*	284	–114.854	18.190	–6.314	<0.000	0.13	Medium
*Tulips–Orchids*	409	34.122	14.824	2.302	0.021	0.01	Small

**Disengagement**							

*Dandelions–Tulips*	339	–69.497	17.354	–4.005	<0.000	0.05	Small
*Dandelions–Orchids*	284	–80.145	18.185	–4.407	<0.000	0.07	Medium
*Tulips–Orchids*	409	10.648	14.821	0.718	0.472	0.00	–

*Interpretation of η^2^: 0.01 – small, 0.06 – medium; 0.14 – large (Cohen, 1988).

The analysis between the tested variables and the sociodemographic characteristics revealed interesting relationships. Age was associated with one significant observation: it correlated with the *Emotional Reactivity* subscale (*r_*S*_* = −0.15, *p* < 0.001), whereas gender revealed more significant observations. Table 8 shows the gender distribution in each cluster and in the general sample. In this study, a significant gender difference was observed: women revealed higher scores in sensitivity and in burnout symptoms.

**TABLE 8:** Analysis of gender distribution among selected clusters and in the general sample.

		**GENDER**		**Total**
		**Women**	**Men**	
CLUSTER	*Dandelions*	16 (3.1%)	89 (17.2%)	105 (20.3%)
	*Tulips*	88 (17.0%)	143 (27.7%)	231 (44.8%)
	*Orchids*	132 (25.6%)	45 (8.7%)	177 (34.3%)
Total		236 (45.7%)	277 (53.7%)	513 (99.4%)

*Three participants (0.6%) did not declare their gender.*

Considering the HSPS subscales, higher scores in women were observed in *Emotional Reactivity*, *Sensing the Subtle*, and *Overstimulation* (Table 9). The results of the Mann–Whitney *U* test show that women and men significantly differ on each HSPS subscale, with large effect size for general HSPS scores (η^2^ = 0.22). Additionally, a Mann–Whitney *U* test revealed that burnout symptoms were significantly higher among women as compared to men, with a small effect size for both *Exhaustion* (η^2^ = 0.03) and *Disengagement*: (η^2^ = 0.02).

**TABLE 9:** Results of the Mann–Whitney *U* test when comparing the groups of women (*n* = 236) and men (*n* = 277).

	**GENDER**	**Mean**	**SD**	**Mann–Whitney *U* test**	***p***	**η*^2^***	Effect size*
HSPS	Women	4.66	0.61	−10.553			
	Men	4.01	0.64		<0.001	0.21	Large
ER	Women	4.68	0.82	−8.730			
	Men	3.96	0.87		<0.001	0.15	Large
StS	Women	4.94	0.71	−5.352			
	Men	4.58	0.80		<0.001	0.06	Medium
OvSt	Women	4.51	0.91	−9.715			
	Men	3.62	0.99		<0.001	0.18	Large
Exhaustion	Women	2.46	0.47	−4.179			
	Men	2.28	0.41		<0.001	0.03	Small
Disengagement	Women	2.42	0.47	−3.194			
	Men	2.29	0.43		0.001	0.02	Small

*Interpretation of η^2^: 0.01 – small, 0.06 – medium; 0.14 – large (Cohen, 1988).

### Moderation Analysis

**FIGURE 4:**
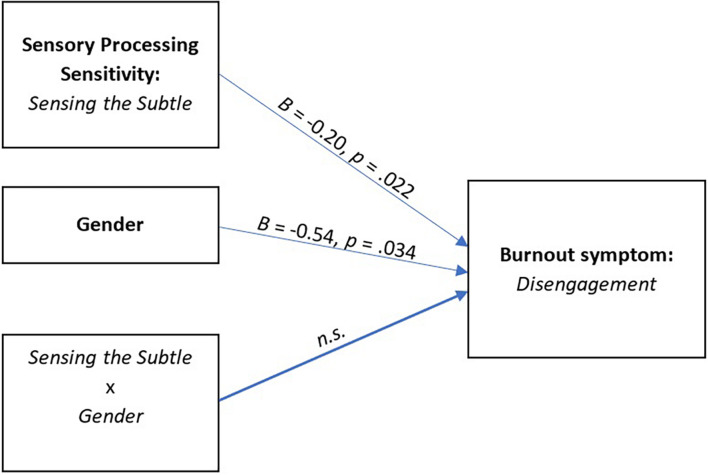
Model of the relations between sensory processing sensitivity (*Sensing the Subtle* subscale) and burnout symptoms (*Disengagement* subscale), moderated by gender (*n.s.*, not significant).

In the next step, moderation analysis was performed to evaluate the role of gender in explaining the relationship between the temperamental characteristics associated with sensory processing sensitivity and burnout symptoms. The type of model 1 (Hayes, 2018) was tested in numerous configurations in which sensitiveness (HSPS, ER, StS, OvSt) was an exposure variable, gender was a moderator, and burnout symptoms (*Exhaustion*, *Disengagement*) were an outcome variable. Figure 4 presents a model [*F*(3,509) = 7.11, *p* < 0.001, *R*^2^ = 0.04] in which gender (*B* = −0.54, *t* = −2.13, *p* = 0.034) and Sensing the Subtle (*B* = −0.20, *t* = −2.30, *p* = 0.022) were shown to be significant variables that explain *Disengagement*, but no significant interaction effect was observed (*B* = 0.08, *t* = 1.54, *p* = 0.125).

This analysis shows that gender is not a significant moderator that could influence the relationship between selected aspects of sensory processing sensitivity and burnout symptoms.

## Discussion

The analyses were focused on exploring burnout syndrome by considering the characteristics of sensory processing sensitivity. Correlation analysis showed that high sensitivity is significantly correlated with burnout symptoms: high sensitivity (general score) is linked with higher levels of exhaustion and disengagement. This relationship is observed in two HSPS subscales: *Emotional Reactivity* (stronger positive correlation) and *Overstimulation* (weaker positive correlation). However, the Sensing the Subtle subscale indicates the opposite pattern: it correlates negatively with exhaustion; this correlation is significant but statistically weak. This result may indicate an interesting aspect in analyzing the role of high sensitivity in the work environment: the ability to perceive details and nuances may protect against employee exhaustion. An employee’s ability to analyze more stimuli, both external and internal, may help them better understand their situation, thus resulting in more reflective and reasonable decisions. These research directions seem to have a potential for further analysis and verification. Secondly, this is the only sensitivity characteristic that demonstrates another pattern in relation to burnout syndrome; thus, it may indicate the advantageous effect of sensitivity. It is also important to emphasize that the StS subscale is weaker than the other HSPS subscales, so its relationships with burnout symptoms needs further analysis and confirmation.

The results of multiple regression analysis imply that high sensitivity is a significant predictor of burnout syndrome. It is especially linked with exhaustion, for which the amount of explained variance and beta coefficients indicate stronger associations with sensitivity. In both tested models (for exhaustion and disengagement), two aspects of high sensitivity were significant, *Emotional Reactivity* (ER) and *Sensing the Subtle* (StS), which consequently demonstrate opposite effects: while ER seems to increase burnout symptoms, StS seems to have the opposite influence. In both models, *Overstimulation* (OvSt) was not shown to be a significant predictor of burnout symptoms. Thus, it may be concluded that high sensitivity may increase susceptibility to burnout symptoms, but it is linked mainly to one aspect of HSP, *Emotional Reactivity*, which is the strongest predictor of both exhaustion and disengagement. Considering the effects of the ability to perceive nuances and subtleties, *Sensing the Subtle* may be seen as a factor that protects against burnout symptoms.

In previous studies using the *Highly Sensitive Person Scale*, latent class analyses consequently suggested a three-factor solution which defined groups of *Dandelions*, *Tulips* and *Orchids* (Lionetti et al., 2018). This may suggest that the HSPS is reliable in culturally diverse samples and remains the most direct attempt to measure the level of sensitivity (May et al., 2020). In the presented study, we have confirmed three clusters of sensitivity whose reactivity, ability to perceive subtleties and nuances, and tendency to be overstimulated characteristics differ: low (*Dandelions*), medium (*Tulips*) and high (*Orchids*). *Orchids* show the highest scores on one general and three specific characteristics of HSP. *Dandelions* demonstrate the lowest scores on general HSPS and the subscales of *Emotional Reactivity* and *Overstimulation*; the *Tulips* are ‘in the middle.’ The results show only one insignificant pair of comparisons: the results of *Dandelions* and *Tulips* in the *Sensing the Subtle* subscale. When the differences in burnout symptoms between the distinguished sensitivity clusters are examined, it may be assumed that the group which has significantly lower levels of exhaustion and disengagement also has the lowest scores in sensitivity, i.e., *Dandelions*. The two other groups, *Tulips* and *Orchids*, differ in the level of exhaustion but reveal similar levels of disengagement.

The obtained data indicate a similar sample distribution of the three groups of sensitivity as was reported by Lionetti et al. (2018): a low-sensitive group – 21% (Lionetti et al., 2018: 29%); medium-sensitive – 45% (Lionetti et al., 2018: 40%); and high-sensitive – 34% (Lionetti et al., 2018: 31%). It may be concluded that the medium-sensitivity group is the most representative; however – quite surprisingly – the second largest group was the high-sensitivity group; the lowest sensitivity group is the smallest group. This means that highly sensitive employees do not form a marginal group but may represent 1/3 of all workers. Considering the characteristics of this group and the correlations between high sensitivity and burnout symptoms, it seems that there should be a focus on understanding the needs and resources of highly sensitive employees.

When analyzing the structure of the *Highly Sensitive Person Scale*, we tested the three-factor structure of HSPS and revealed nearly the same structure as was reported by Smolewska et al. (2006). However, when analyzing the content of the questions of each HSPS component and labeling the components, we decided to refer to the DOES model of sensitivity. The inspiration for changing the components’ labels came from doubt in the second component, which in Smolewska et al. (2006) is defined as *Aesthetic Sensitivity*. This component refers to perceiving subtleties and nuances; a rich and complex inner life; conscientiousness; and being moved by the arts and music. In our opinion, *Aesthetic Sensitivity* has connotations that are too narrow, whereas the StS subscale items refer to broader aspects of depth of processing and the ability to sense subtleties, which are the core elements of the DOES model of sensitivity (Aron et al., 2012; Aron, 2013). Therefore, when labeling the principal components we refer to the DOES model, which includes *depth of processing* (D), *overstimulation* (O), *emotional reactivity* (E), and *sensing the subtle* (S). Nevertheless, as the structure of HSPS components is nearly identical to the previous studies (Smolewska et al., 2006), we can conclude that the components extracted here refer directly to those defined by Smolewska et al. (2006): the component labeled as *Emotional Reactivity* (ER) refers to “Ease of Excitation” (EOE); *Sensing the Subtle* (StS) refers to “Aesthetic Sensitivity” (AES); and *Overstimulation* (OvSt) refers to “Low Sensory Threshold” (LST).

Interestingly, depth of processing seems to be located somewhere between the extracted components or it combines all the selected components as it refers to empathy, conscientiousness, rich imagination and inner life, thoughtfulness, and awareness of consequences. Depth of processing is associated with the “pause-to-check approach,” which enables greater consideration or reflection (Aron, 2013). Analyzing the HSPS items seems to show that deep processing is linked with both emotional reactivity and the ability to perceive nuances, but depth of processing is a broader construct than this. In our opinion, this aspect of high sensitivity should be extended and detailed in another measure of the HSP construct.

Following previous findings that indicated higher levels of sensitivity among women (e.g., Jonsson et al., 2014; Şengül-l̇nal and Sümer, 2020), this study explores gender differences. The presented results confirm this tendency: women demonstrated higher scores on the general HSP scale and on each of the HSP subscales. Women revealed higher levels of all aspects of sensitivity (*Emotional Reactivity*, *Sensing the Subtle*, and *Overstimulation*). Women also demonstrated significantly higher levels of burnout symptoms. This outcome may have an important meaning in the context of improving well-being. The presented results may indicate why, in many studies on burnout, women are more exhausted at work. This has often been linked with sociodemographic characteristics, e.g., the necessity to take care of children and the problem of conflicting roles. This study shows that another aspect that should be considered in explaining the problem of burnout among women employees is sensitivity as an individual disposition. We did not found a moderating effect of gender in explaining the relationship between sensory processing sensitivity and burnout symptoms. However, referring to the results of Nocentini et al. (2018), it may be supposed that such moderating effect may be important in intervention programs against burnout.

One important aspect which is not precisely reflected on the *Highly Sensitive Person Scale* is depth of processing, which may be described in terms of reflexivity, intuition, wisdom resulting from caution, deliberate behavior, and the ability to take others’ perspectives (Aron et al., 2012; Aron, 2013; Acevedo et al., 2014, 2017). Highly sensitive persons are described as “royal advisors” (Aron, 2013), which may create an attractive identity for those who perceive themselves as highly sensitive. In view of such HSP characteristics, there are some advantages in identifying oneself with HSP. Firstly, it definitely strengthens the self-esteem of people who – until they became acquainted with the concept of HSP – perceived themselves negatively in the contexts of maladjustment, shyness, and low sociability, all of which might lead to unfavorable consequences, e.g., low self-esteem. If high sensitivity is described not in terms of disadvantages but in terms of specific resources, this enables highly sensitive persons to feel unique, to gain deeper self-knowledge, and to be willing to change their environment or circumstances according to their needs. This is particularly important in rigid organizations, which are characterized by a hierarchical structure, defined tasks, procedures, and regulations, and in which changes are difficult to implement.

Many psychological theories remain unknown to the wider public, despite the fact that they explain the essential mechanisms of individual and social functioning. This may happen due to dry scientific language which is not appealing to non-psychologists. An invaluable aspect of Aron and Aron’s HSP concept (1997) is its application value: it explains how specific dispositions may influence mental and emotional states and the possible consequences of high sensitiveness in favorable and unfavorable environments. This concept provides specific ‘guidelines’ for coping strategies in working environments and has practical implications for self-psychotherapy tips, e.g., reformulation of one’s own way of reacting to changes, assessing new situations, changing the external context, and using support, self-care, and enriching strategies in order to avoid overstimulation (Aron, 2012). Aron and Aron (1997); Aron et al. (2012), Acevedo et al. (2014), and Greven et al. (2019) indicate associations between HSP and temperamental and personality characteristics that indicate higher reactivity to the conditions of the surrounding environment. According to Aron and Aron (1997), high sensitivity is especially associated with the behavioral inhibition system (BIS; Gray, 1982) and is responsible for the ‘pause-to-check’ function. In the revised Reinforcement Sensitivity Theory (RST; Gray and McNaughton, 2000), it was assumed that BIS is activated by both positive and negative stimuli that accordingly activate the Behavioral Approach System (BAS) and the Flight, Fight and Freezing System (FFFS). In the context of the revised RST, it may be assumed that HSP is associated with higher sensitivity to both positive and negative environmental stimuli. This perspective may be of great significance in understanding the possible consequences of HSP characteristics in work environments. A highly sensitive person may not only be prone to overstimulation but may also be predisposed to reacting more strongly to positive stimuli that have great meaning in motivational processes.

From both an individual and an organizational perspective, it is particularly important to explore how to use this knowledge on high sensitivity in work environments to enhance well-being at work and prevent burnout among HSPs. When analyzing the practical implications of these findings, it should be emphasized that high sensitivity should not be simplified and treated as a direct and simple predictor of burnout. Some aspects of HSP are positively linked with burnout (ER), whereas others are linked negatively (StS). As compared to a non-sensitive individual, for a highly sensitive person unfavorable working conditions, such as work overload, conflicts, insufficient autonomy, etc., can have ‘a double negative effect’ and may result in further negative consequences. Significant relations between emotional reactivity and burnout symptoms, especially with exhaustion, should be considered when analyzing the further possible consequences of burnout (e.g., reduced well-being, depression and other health problems) and making recommendations for employee well-being. In this context, proper work design with a favorable work environment, adequate workload and sufficient support are of the highest significance. On the individual level, regulating strategies aimed at reducing emotional/psychophysiological arousal, awareness of the first symptoms of being overloaded, and the ability to respond to signs of overstimulation by taking breaks and limiting sources of distress may contribute to developing adequate and functional solutions. Evers et al. (2008) suggest that exhaustion, tiredness, irritation or frustration are natural reactions to overstimulation: they are not pathological. These researchers indicate that balance can be regained by practicing yoga, meditation, physical recreation, and sports. It is especially significant to develop knowledge on one’s dispositions and to increase one’s sense of effective coping and controllability. This type of intervention not only allows highly sensitive people to function effectively in the work environment; it also generates profits in the workplace related to the specificity of highly sensitive persons’ sensory processing, their ability to capture subtleties, and their reflective approach to problems.

### Limitations

In the presented study, only general company profile information was collected. Future studies may focus on analyses of different types of jobs, exploring how specific tasks and the work environment influence the relationships between SPS and burnout symptoms. This would make is possible to determine whether the associations between SPS and burnout symptoms are universal or are also influenced by work context. These analyses could be extended to exploring other aspects of work, such as salary, sick days, promotions, or awards. Furthermore, it would be particularly important to investigate other individual outcomes of highly sensitive persons in their work environment. This should not be limited to negative consequences (e.g., health complaints, anxiety, depressive symptoms); it should also include positive outcomes, such as higher creativity, greater decision accuracy or better teamwork, thanks to higher reflectiveness and empathy which are linked with high SPS.

A deeper understanding of the mechanisms underlying the association between high sensitivity and burnout would be provided by additional analyses of, e.g., the temperamental variables that determine excitability and reactivity, emotional states (especially anxiety and depression), neuroticism, emotional style, reflectivity, resilience, and coping strategies. As has been indicated by other researchers (Acevedo et al., 2018; Greven et al., 2019), HSP should be further explored due to its association with other psychological constructs.

The instruments used in the study have satisfactory reliability coefficients, but the *Sensing the Subtle* subscale proved to be weak as it had a Cronbach alpha of 0.61. This indicates the need for further research on developing an instrument dedicated to this aspect of high sensitivity. A more reliable instrument would make it possible to determine whether special sensitivity to nuances and subtleties is a significant factor that reduces the risk of burnout.

## Conclusion

Sensory processing sensitivity reveals significant relationships with burnout symptoms, i.e., higher emotional reactivity is linked to increased exhaustion and disengagement from work, while greater ability to sense the subtle shows the opposite effect and may be a protective factor against exhaustion. Thus, it seems particularly important to differentiate specific aspects of SPS which may have opposite effects on burnout symptoms. These results may indicate that two regulatory strategies may be useful for highly sensitive employees to reduce burnout: (1) dealing with emotional reactivity by regulating psychophysiological arousal; (2) strengthening the ability to sense the subtle by means of cognitive training and mindful attentional awareness.

### Future Directions

Further research on the association between sensing subtleties and nuances, depth of processing and burnout syndrome is needed; this may be a very interesting perspective in which some aspects of sensitivity may be considered as protective factors against burnout symptoms. To explore this area, however, a more sensitive instrument for these aspects of sensitivity should be developed. In further research it could be valuable to include sensory processing sensitivity as an individual disposition that may significantly influence employees’ outcomes.

## Data Availability Statement

The raw data supporting the conclusions of this article will be made available by the authors, without undue reservation.

## Ethics Statement

The studies involving human participants were reviewed and approved by the Research Ethics Committee of the Institute of Applied Psychology, Jagiellonian University in Kraków. Written informed consent for participation was not required for this study in accordance with the national legislation and the institutional requirements.

## Author Contributions

KG and BG substantially contributed to the conception and design of the work, acquisition, analysis, interpretation of data, drafting the work and revising it critically, final approval of the version to be published, and agreed to be accountable for all aspects of the work.

## Conflict of Interest

The authors declare that the research was conducted in the absence of any commercial or financial relationships that could be construed as a potential conflict of interest.

## Publisher’s Note

All claims expressed in this article are solely those of the authors and do not necessarily represent those of their affiliated organizations, or those of the publisher, the editors and the reviewers. Any product that may be evaluated in this article, or claim that may be made by its manufacturer, is not guaranteed or endorsed by the publisher.
